# Poly (dopamine) coated superparamagnetic iron oxide nanocluster for noninvasive labeling, tracking, and targeted delivery of adipose tissue-derived stem cells

**DOI:** 10.1038/srep18746

**Published:** 2016-01-05

**Authors:** Naishun Liao, Ming Wu, Fan Pan, Jiumao Lin, Zuanfang Li, Da Zhang, Yingchao Wang, Youshi Zheng, Jun Peng, Xiaolong Liu, Jingfeng Liu

**Affiliations:** 1 The United Innovation of Mengchao Hepatobiliary Technology Key Laboratory of Fujian Province, Mengchao Hepatobiliary Hospital of Fujian Medical University, Fuzhou 350025, P.R. China; 2 The Liver Center of Fujian Province, Fujian Medical University, Fuzhou 350025, P.R. China; 3 Department of Hepatobiliary Surgery, Fuzong Clinical College, Fujian Medical University, Fuzhou 350001, P.R. China; 4 Academy of Integrative Medicine, Fujian University of Traditional Chinese Medicine, Fuzhou 350122, P.R. China; 5 Liver Disease Center, The First Affiliated Hospital of Fujian Medical University, Fuzhou 350007, P.R. China

## Abstract

Tracking and monitoring of cells *in vivo* after transplantation can provide crucial information for stem cell therapy. Magnetic resonance imaging (MRI) combined with contrast agents is believed to be an effective and non-invasive technique for cell tracking in living bodies. However, commercial superparamagnetic iron oxide nanoparticles (SPIONs) applied to label cells suffer from shortages such as potential toxicity, low labeling efficiency, and low contrast enhancing. Herein, the adipose tissue-derived stem cells (ADSCs) were efficiently labeled with SPIONs coated with poly (dopamine) (SPIONs cluster@PDA), without affecting their viability, proliferation, apoptosis, surface marker expression, as well as their self-renew ability and multi-differentiation potential. The labeled cells transplanted into the mice through tail intravenous injection exhibited a negative enhancement of the MRI signal in the damaged liver-induced by carbon tetrachloride, and subsequently these homed ADSCs with SPIONs cluster@PDA labeling exhibited excellent repair effects to the damaged liver. Moreover, the enhanced target-homing to tissue of interest and repair effects of SPIONs cluster@PDA-labeled ADSCs could be achieved by use of external magnetic field in the excisional skin wound mice model. Therefore, we provide a facile, safe, noninvasive and sensitive method for external magnetic field targeted delivery and MRI based tracking of transplanted cells *in vivo*.

Pluripotent adipose tissue-derived stem cells (ADSCs) have the advantages of being abundant, easy obtain and immuno-regulatory^1^, which can differentiate into adipocyte, chondrocyte and osteocyte lineages, as well as the neurons and hepatocytes^2^. Thus, ADSCs transplantation has attracted great attention as a new therapeutic tool for treating various diseases ranging from cardiovascular diseases, liver and renal diseases to neurological diseases^2^,^3^,^4^,^5^. However, these cell treatments remain experimental, and the safety and therapeutic mechanisms of ADSCs-based therapy are largely unclear. Better understanding of the cell fate and homing *in vivo* after transplantation will significantly accelerate the clinical translation of stem cell therapy^6^.

Various kinds of imaging modalities, such as fluorescence imaging (FLI), bioluminescence imaging (BLI)^7^,^8^, positron emission tomography (PET), single photon emission computered tomography (SPECT)^9^,^10^, and magnetic resonance imaging (MRI)^11^,^12^,^13^,^14^, have been used to track the stem cells after transplanting *in vivo*. Nevertheless, the usage of FLI and BLI have been limited due to low tissue penetrating depth as well as photo attenuation and scattering^15^,^16^; on the other hand, both PET and SPECT have the disadvantage of being ionizing radiation^17^. Among these imaging modalities, MRI is an ideal tool for stem cell tracking because of its inherent soft-tissue contrast, high perforated depth and non-ionizing radiation^11^, and it has been successfully used for clinical evaluation of stem cell therapies^12^,^13^. MRI tracking of stem cells, which is often performed by labeling the cells with contrast agents to distinguish them from the endogenous tissues^11^,^18^,^19^, can monitor the cell homing and the therapeutic effects of disease treatment non-invasively. Although the commercial contrast agents, such as dextran-coated Endorem and carboxydextran-coated Resovist, have been widely used for labeling stem cells^20^,^21^, they still suffer from low sensitivity because of the low relaxivity of individual superparamagnetic iron oxide nanoparticles (SPIONs) in these contrast agents. Therefore, extensive efforts have been devoted to the development of novel SPIONs with high efficiency and sensitivity in the field of stem cell labeling. For example, Kim *et al.* fabricated SPIONs coupled with 2-aminoethyl-trimethyl ammonium as a simple and rapid stem cell labeling agent for MRI tracking^22^. Andreas *et al.* used citrate-coated SPIONs for efficient magnetic stem cell labeling^23^. Although the imaging sensitivity has been extensively improved in these studies, the biocompatibility and potential influence on the inherent characteristics of stem cells are needed to be further studied. Meanwhile, targeted delivery of the stem cells to designated location is still a big challenge in stem cell-based therapies. Therefore, integrating the imaging and targeted delivery functions together in a single nanoplatform would significantly accelerate the clinical translation of stem cell therapy.

Previously we have succeeded in the synthesis of a core-shell nanocomposite of clusters of superparamagnetic iron oxide nanoparticles coated with poly (dopamine) (SPIONs clusters@PDA) as a highly sensitive and biocompatible magnetic resonance imaging (MRI) contrast for cancer cells^24^. The collective properties of individual SPIONs in the cluster core of this nanocomposite can provide higher sensitivity by increasing the relaxivity of the incorporated contrast agents through decreasing the molecular tumbling rates, and the PDA shell endow the nanocomposite with colloidal stability and low cytotoxicity for cell labeling. In the current study, we have established a mouse model with liver injury induced by carbon tetrachloride (CCl_4_), and investigated the homing capability and therapeutic effects of SPIONs cluster@PDA labeled ADSCs after liver injury *in vivo*. To the best of our knowledge, this is the first study associated with the MRI tracking of ADSCs in the liver injury with a high r_2_ relaxivity. In addition to the high MRI contrast enhancing ability, the SPIONs cluster@PDA nanocomposites have also been demonstrated to respond rapidly to external magnetic field gradients in our previous work. Followed by this advantage, we further established a mouse model of excisional skin wound to investigate the targeted guiding of cell homing to the wound by an external magnetic field and subsequently the curative effects of the SPIONs cluster@PDA labeled ADSCs.

## Results and Discussion

### Characterization the morphology of the SPIONs cluster@PDA

**Figure 1:**
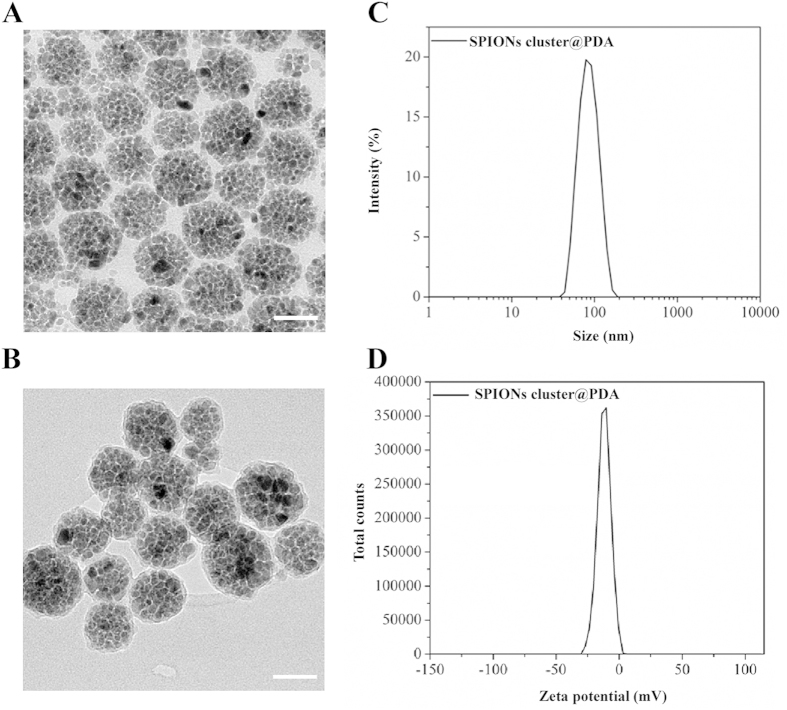
TEM image of SPIONs cluster (A) and SPIONs cluster@PDA (B). Scale bar: 50 nm.

The morphology of the prepared SPIONs cluster@PDA was determined by TEM measurements. Fig. 1A illustrates the representative TEM pictures of obtained nanoparticles, which clearly revealed that the as-prepared products were mainly composed of spherical SPIONs cluster with the diameter ranging from 40–100 nm. A coating layer of PDA on the surface of SPIONs nanocluster with an approximately 4 nm thickness can be found in Supplementary Fig. S1 online. DLS studies (Fig. 1A) reveal that the average hydrodynamic size of SPIONs cluster@PDA in aqueous solution was 81.5 nm, which is consistent with the size by TEM. Furthermore, the PDI was determined to be 0.056, indicating a relatively good dispersion distribution. The zeta potential distribution of SPIONs cluster@PDA in aqueous solution is relatively narrow (zeta deviation: 5.29 mV) and centered at −11.9 mV.

Meanwhile, the weight loss of SPIONs cluster@PDA and SPIONs cluster has been determined to be 49.19 wt% and 18.8 wt% through thermogravimetric analysis respectively^24^. Therefore, the amount of PDA on SPIONs cluster is 30.39 wt% which can be calculated by subtracting the weight loss of SPIONs cluster from weight loss of SPIONs cluster@PDA. Furthermore, the Fe content in SPIONs cluster@PDA was determined to be 36.4 wt% according to the colorimetric method.

### Cytotoxic evaluation of the SPIONs cluster@PDA to ADSCs

**Figure 2:**
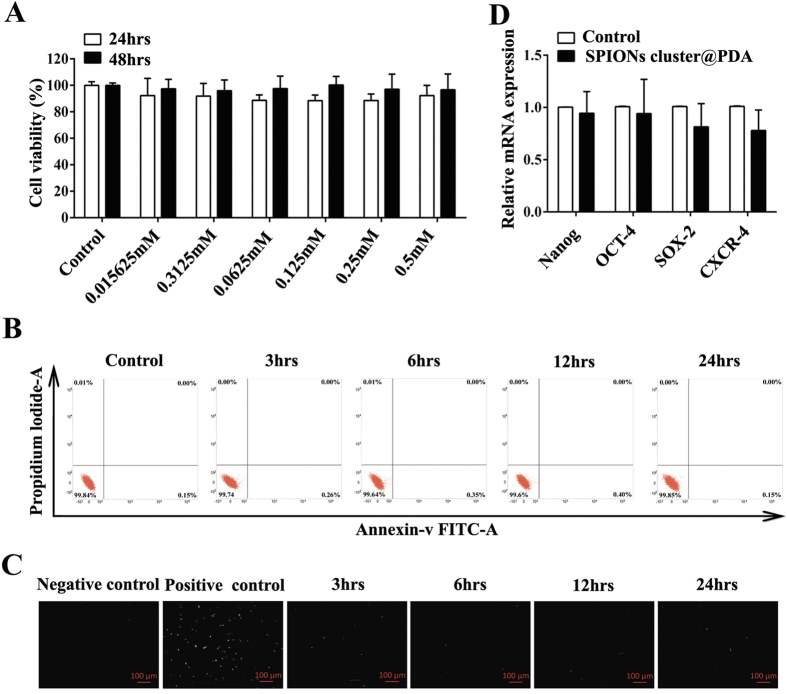
The effects of SPIONs cluster@PDA on cell viability, apoptosis and gene expression of ADSCs. (**A**) Cell viability of ADSCs cultured with different concentrations of SPIONs cluster@PDA for 24 hrs and 48 hrs. Cell apoptosis was evaluated by both flow cytometry (**B**) and TUNEL assay (**C**) after incubation with 0.25 mM SPIONs cluster@PDA for 3 hrs, 6 hrs, 12 hrs and 24 hrs, respectively. Positive control: ADSCs treated with recombinant DNase I (300 U/ml in 50 mM Tris-HCl, pH 7.5, supplemented with 1 mg/ml BSA) for 10 min at 25 °C to induce cell apoptosis according to the manufacture’s protocol. Scale bar, 100 μm. (**D**) The relative gene expression of Nanog, OCT-4, SOX-2 and CXCR-4 of after incubation with 0.25 mM SPIONs cluster@PDA for 24 hrs.

It is generally considered that iron oxide is non-toxic to cells, since it can be degraded and utilized by cells via physical iron metabolism pathway^25^,^26^. However, the safety of SPIONs is composition dependent^27^, and several groups have recently reported that the free Fe^3+^ ions would induce the adverse effects on stem cells^27^,^28^,^29^,^30^. Here, the cell viability of SPIONs cluster@PDA-labeled ADSCs was evaluated using CCK 8 assay, and the results demonstrated that there was no obvious change in cell proliferation after the labeling; even at a high dose of 0.5 mM, the cells remain more than 90% viable after 48 hrs incubation (Fig. 2A). We next investigated the cell apoptosis of ADSCs to further confirm the non-cytotoxicity of SPIONs cluster@PDA, which was determined by flow cytometry using Annexin-V-FITC/PI staining. As shown in Fig. 2B, nearly all the cells were localized in the lower left quadrant with more than 99% of the viable cells after incubating with SPIONs cluster@PDA (0.25 mM) for 24 hrs, which demonstrates the very low cytotoxicity of SPIONs cluster@PDA. In addition to the Annexin-V-FITC/PI staining methods, TUNEL assay was also used to evaluate the cell apoptosis after treated with SPIONs cluster@PDA through an inverted fluorescence microscope, as the apoptosis cells will emit green fluorescence. As shown in Fig. 2C, it could hardly see any apoptotic cells (green fluorescence cells) after incubated with SPIONs cluster@PDA (0.25 mM) within the incubtion time ranging from 3 hrs to 24 hrs.

Transcription factors, including Nanog, SOX-2, and OCT-4, have been proposed to play an important role in the maintenance of the pluripotent state of stem cells. Therefore, we evaluate the influence of SPIONs cluster@PDA on the expression of these transcription factors using quantitative real-time PCR analysis. As shown in Fig. 2D, the expression of these stem cell makers was similar with the untreated ADSCs (control), suggesting no influence of SPIONs cluster@PDA on cell stemness.

CXCR-4 have been reported to be one of chemokine receptor which can mediate stem cell homing, and Chen *et al.* reported that iron-based magnetic nanoparticles could actively increase the expression of chemokine receptor CXCR-4 in bone-marrow-derived MSCs and improve homing of MSCs to the injury sites^31^,^32^,^33^. Therefore, we also attempt to determine whether SPIONs cluster@PDA could actively regulate the expression of the CXCR-4 on ADSCs using quantitative real-time PCR. However, our results showed that there was no significant increase of the CXCR-4 expression after the SPIONs cluster@PDA labeling (Fig. 2D).

### Stem Cell surface marker expression and the multipotent differentiation ability of the SPIONs cluster@PDA-labeled ADSCs

**Figure 3:**
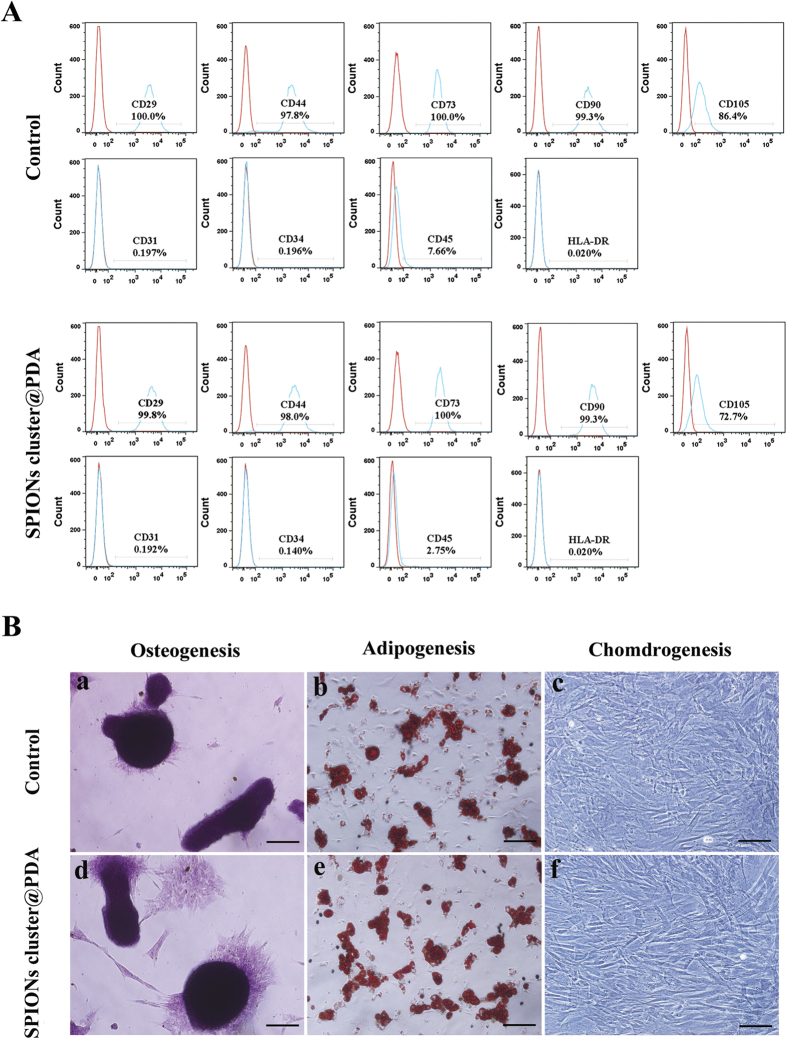
Surface stem cell marker expression and the multiple-differentiation potentials of SPIONs cluster@PDA-labeled ADSCs. (**A**) Flow cytometry analysis of the surface stem cell marker expression of the labeled and unlabeled ADSCs. Red lines indicate the negative control, while blue lines indicate the expression of the surface biomarkers. (**B**) Multi-differentiation potentials of the labeled and unlabeled ADSCs toward osteogenesis (Alizarin Red S staining; Scale bar, 50 μm), adipogenesis (Oil Red O staining; Scale bar, 50 μm), and chondrogenesis (toluidine blue staining; Scale bar, 50 μm).

In order to further study the influence of SPIONs cluster@PDA on ADSCs, stem cell surface markers were examined by flow cytometric analysis after being labeled with SPIONs cluster@PDA. As shown in Fig. 3A, both the labeled and unlabeled ADSCs were positive for CD29, CD44, CD73, CD90 and CD105, and negative for CD31, CD34, CD45 and HLA-DR. These results further confirm that there are no obvious influences of the SPIONs cluster@PDA labeling to the properties of ADSCs.

The effects of nanoparticles on the differentiation potential of ADSCs are a crucial concern for developing stem cell tracking. In this respect, we further studied the influences of SPIONs cluster@PDA on differentiation of ADSCs into osteogenic, adipogenic and chondrogenic mesodermal lineages (three typical characteristics of ADSCs), and the results are shown in Fig. 3B. In control group, the red mineralized nodules, lipid droplets and chondrocyte-like cells (lavender) were positively stained by the Alizarin Red S, Oil Red O and toluidine blue staining kits, respectively, which indicates that ADSCs can be successfully differentiated into osteogenic, adipogenic and chondrogenic lineages in the presence of differentiation supplements. After incubation with 0.25 mM SPIONs cluster@PDA, the number of mineralized nodules, lipid droplets and chondrocyte-like cells was similar with the control group, suggesting that the SPIONs cluster@PDA nanoparticles do not interfere the osteogenic, adipogenic, or chondrogenic lineage differentiation abilities of the labeled ADSCs.

### Uptake of the SPIONs cluster@PDA into ADSCs

**Figure 4:**
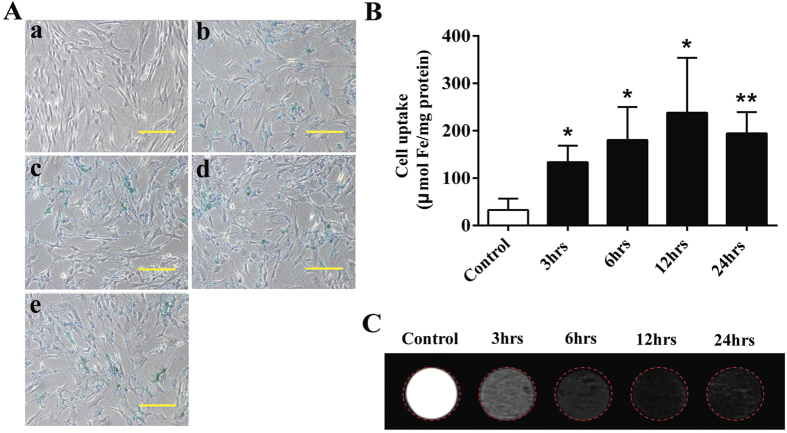
Uptake of SPIONs cluster@PDA into ADSCs. (**A**) Intracellular SPIONs cluster@PDA in ADSCs was detected by Prussian blue staining after the incubation with SPIONs cluster@PDA (a: control; b: for 3 hrs; c: for 6 hrs; d: for 12 hrs; e: for 24 hrs). Scale bar: 100 μm. (**B**) Intracellular iron content was quantificationally determined after incubated with 0.25 mM SPIONs cluster@PDA for 3 hrs, 6 hrs, 12 hrs and 24 hrs, respectively. (**C**) T_2_-weighted phantom images of ADSCs incubated with 0.25 mM SPIONs cluster@PDA for 3 hrs, 6 hrs, 12 hrs and 24 hrs, respectively.

For a contrast agent of stem cell tracking, efficient internalization by stem cells is of great importance. Thereby, Prussian blue staining was used to qualitatively determine the iron content inside the ADSCs. As shown in Fig. 4A, comparing with the unlabeled cells, blue granules can be observed in the cells incubated with SPIONs cluster@PDA for 3 hrs, clearly proving that the nanocomposites could be efficiently uptake by ADSCs. Specifically, a significant increase of the amount of granules was observed along with a concomitant increase of the incubation time from 3 hrs to 12 hrs.

Subsequently, the cellular uptake of SPIONs cluster@PDA was further explored by the quantification of cellular iron content determined by a colorimetric method. After ADSCs was incubated with or without 0.25 mM SPIONs cluster@PDA for 3 hrs, 6 hrs, 12 hrs and 24 hrs, respectively, the cells were collected and measured. As shown in Fig. 4B, compared with those unlabeled cells (control group, 32 μmol mg^−1^ protein), the iron concentration of ADSCs was significantly increased to 133 μmol mg^−1^ protein after the incubation of SPIONs cluster@PDA for 3 hrs, and the cellular iron concentration could be further increased to 180 and 238 μmol mg^−1^ protein with the incubation time extended to 6 hrs and 12 hrs, respectively. However, after the incubation for 24 hrs, the cellular iron concentration showed a slight decrease compared to those incubated with nanocomposites for 12 hrs, which implying that the maximum cellular uptake had reached after incubating with SPIONs cluster@PDA for 12 hrs.

Due to the highly efficient cellular uptake of SPIONs cluster@PDA, we next used the SPIONs cluster@PDA composite for MR imaging of ADSCs. As shown in Fig. 4C, compared with unlabeled cells, negative enhancement of the MRI signal was observed in labeled cells, and the T_2_-weighted images of the labeled cells became darker along with increased incubation time ranging from 3 hrs to 12 hrs. However, after being incubated with SPIONs cluster@PDA for 24 hrs, the labeled cells did not exhibit further reduced signal, ascribing to the cellular uptake had reached the maximum level after incubating with SPIONs cluster@PDA for 12 hrs.

### *In vivo* tracking of the SPIONs cluster@PDA labeled ADSCs in a CCl_4_ induced liver injury model

**Figure 5:**
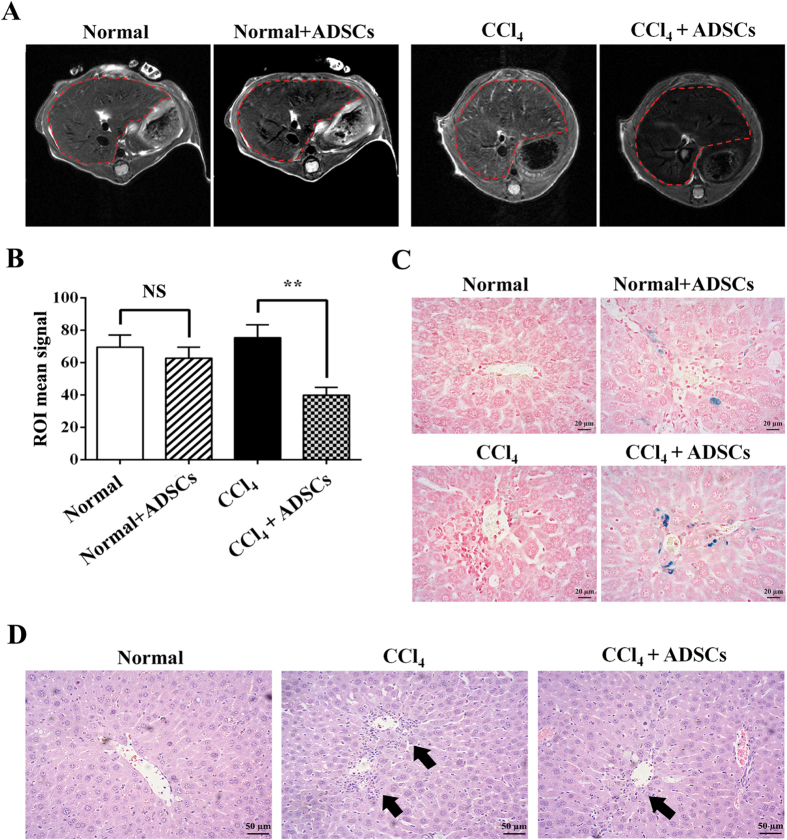
MRI tracking of the SPIONs cluster@PDA labeled ADSCs after transplantation in a CCl_4_ induced liver injury murine model *in vivo*. (**A**) *In vivo* imaging of the SPIONs cluster@PDA-labeled ADSCs homing to injured liver after transplantation for 1 hr. (**B**) The average gray values of mice liver (ROI as indicated in part A). (**C**) Prussian blue staining of the liver after transplantation with SPIONs cluster@PDA-labeled ADSCs for 1 hr. (**D**) Histo-pathological assessment of the injured liver induced by CCl_4_. Scale bar: 20 μm.

Because of the potential differentiation of ADSCs into hepatocytes, as well as their good immunoregulation and inherent chemotactic ability to home to the injured liver sites, ADSCs transplantation has been widely used to treat various liver diseases such as liver failure^34^,^35^,^36^, liver cirrhosis^37^, and non-alcoholic liver diseases^38^. However, it is very important to successfully track the delivery of transplanted ADSCs to the injured liver with a noninvasive imaging modality in clinical application. Since the MR imaging of SPIONs cluster@PDA-labeled ADSCs has been achieved *in vitro*, we further conducted *in vivo* experiments in a mouse model with liver injury induced by CCl_4_ to track the migration of SPIONs cluster@PDA-labeled ADSCs toward the injured liver. T_2_-weighted MR images of mice liver (Fig. 5A) were acquired pre- and post- tail intravenous injection of 1 × 10^6^ SPIONs cluster@PDA-labeled ADSCs. A slight increased T_2_ signal of normal liver (without CCl_4_ treated) of mice could be observed after receiving injection of the labeled ADSCs for 1 hr (Fig. 5A), which indicates some labeled ADSCs deliver to the normal liver. Notably, in contrast with the liver of normal mice received the injection of labeled ADSCs, the enhancement of the T_2_ signal could be clearly observed in CCl_4_-injured liver after injection for 1 hr (Fig. 5A). The increase in MRI signal was quantified by measuring the gray values of region of interest (ROI) of the liver in Fig. 5B. The CCl_4_-injured liver showed a much lower gray values after ADSCs injection, while there were no significant changes of gray values in normal liver model which also had been injected with ADSCs. This result indicates a rapid and efficient accumulation of ADSCs in the injured liver compared with the normal liver.

To further demonstrate the increased accumulation of labeled ADSCs inside the injured liver, the liver tissues of the treated mice were collected and the homing ADSCs was examined by the Prussian blue staining. As shown in Fig. 5C, compared with normal mice without ADSCs injection, some blue granules could be clearly observed around the central vein of normal liver, which means SPIONs cluster@PDA-labeled ADSCs can home to the liver, even without any injury. However, after the liver injury induced by CCl_4_, there are significant increased blue granules appearing in the liver. This result is consistent with the result of MR imaging *in vivo*, and demonstrates that the SPIONs cluster@PDA-labeled ADSCs have the homing capacity to the CCl_4_-injured liver, and therefore provides a noninvasive method for tracking the homing of ADSCs to the injured sites.

In the light of the substantial homing of SPIONs cluster@PDA-labeled ADSCs to the injured liver, we next explored whether the injection of labeled ADSCs could promote recovery of CCl_4_-injured liver by using the hepatic-pathological examination. As shown in Fig. 5D, no incidence of injury was observed in the central vein of liver tissues of normal mice, while typical inflammatory infiltration (black arrow in Fig. 5D) was clearly shown around the central vein of injured liver induced by CCl_4_, which means that the liver injury model is successfully established by the administration of CCl_4_. Thus, we used this model to exam the therapeutic effects of labeled ADSCs. Considerably, compared with those without ADSC injection, less incidences of inflammatory infiltration were appeared around the liver central vein of treated mice after tail intravenous injection of the SPIONs cluster@PDA-labeled ADSCs for 24 hrs, implying that the labeled ADSCs could promote the repair of injured liver. These results are in well consistent with the previous studies that unlabeled ADSCs can ameliorate the liver injuries induced by CCl_4_ in rodent model^39^,^40^,^41^,^42^. Hence, above data indicate the therapeutic effects of ADSCs on liver injures cannot be affected by the labeling of SPIONs cluster@PDA nanoparticles, and also proved the good biocompatibility of these nanoparticles.

The liver also played important roles in the clearance of administrated cells or nanoparticles. To confirm the homing and distribution of ADSCs for repair but not due to the clearance effects, we further tracked the fluorescence tag EGFP stably expressed ADSCs in injured liver model. After the intravenous transplantation of EGFP-ADSCs into the CCl_4_-injured or normal mice for 1 hr, 6 hrs or 24 hrs, the major organs (heart, lung, kidney, spleen and liver) were subsequently harvested and subjected to fluorescence imaging. *in vivo* distribution of fluorescence labeled ADSCs is shown in Supplementary Fig. S2 online. In the normal mice, strong fluorescence signal of ADSCs was detected in the liver and lung after 1 hr of transplantation through tail vein; afterwards, the fluorescence signal in the liver and lung was significantly decreased after 6 hrs of transplantation, and almost gone after 24 hrs of transplantation; conversely, the fluorescence signal in the kidney was continuously increased along with time. This phenomena might be because that the intravenously administered stem cells will be quickly trapped within capillary beds (often in the lungs), and then cleared from circulation by the liver and kidney. In the CCl_4_-induced liver injury model, strong fluorescence signal in the liver and kidney, but not in the lung, was detected after 1 hr of ADSCs transplantation; meanwhile, the fluorescence signal in the liver can be maintained and no obvious fluorescence decrease was observed till 24 hrs. Comparing with the fluorescence signal distributions of normal nice, this is clearly demonstrated that the administrated stem cells were homed into the injured sites, rather than due to the clearance effects of the liver. Interestingly, strong fluorescence signal in the kidney also can be detected after 24 hrs of transplantation; this might be due to the CCl_4_-induced certain kidney injury. These data also suggested the homing and retention of ADSCs in injured liver, which is consistent with the *in vivo* MRI result.

### Improved homing capacity of SPIONs cluster@PDA labeled ADSCs by an external magnetic field

**Figure 6:**
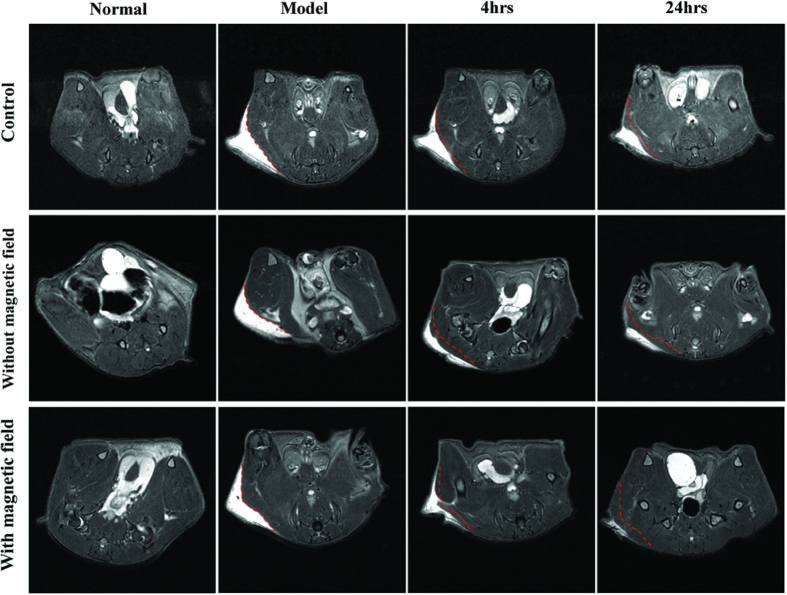
*In vivo* MRI tracking of the homing of SPIONs cluster@PDA-labeled ADSCs toward the injured skin in an excisional skin wound murine model in the presence or absence of an external magnetic field.

Previously, we have shown that the SPIONs clusters@PDA nanocomposites could be rapid response to external magnetic field gradients^24^. Thus, we established a model of excisional skin wound to further verify whether the target-homing capacity of SPIONs cluster@PDA-labeled ADSCs can be improved by the external magnetic field. T_2_-weighted MR images of skin wound were performed pre- and post-injection of the SPIONs cluster@PDA-labeled ADSCs. As shown in Fig. 6, in control group (mice bearing skin wound but without ADSCs injection), a bright T_2_ signal at the region of skin wound was observed, which clearly demonstrated that the damaged area of the skin suffered from edema. Even after 24 hrs, there was still a large area of skin wound with bright T_2_ signal. However, after injection of the SPIONs cluster@PDA-labeled ADSCs for 4 hrs, the bright MRI signal of the wound region was decreased comparing with control group at the same observation time, which demonstrates the SPIONs cluster@PDA-labeled ADSCs could home to the injured skin. More obviously, when an external magnetic field was further applied, the bright MRI signal of the wound region reduced more significantly comparing to the control group or without external magnetic field; the bright MRI signal even nearly disappeared after 24 hrs of the labeled ADSCs injection. These results clearly demonstrated the significantly increased homing capacity and better therapeutic effects of the SPIONs cluster@PDA-labeled ADSCs in the existence of external magnetic field.

### Safety of the SPIONs cluster@PDA labeled ADSCs transplantation

**Figure 7:**
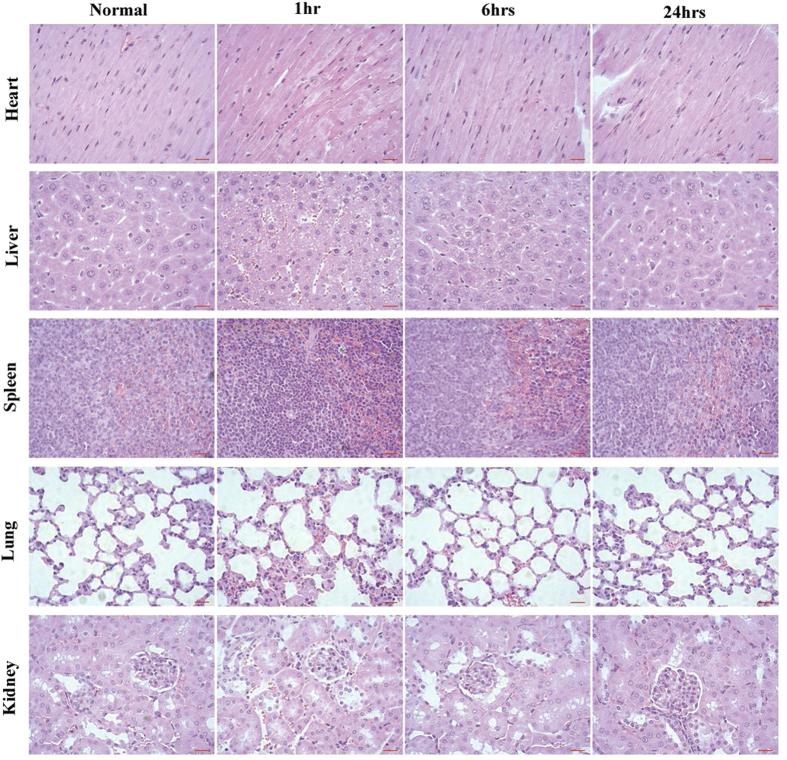
The pathological changes of main organs were evaluated by H&E staining after the transplantation of SPIONs cluster@PDA labeled ADSCs for 1 hr, 6 hrs and 24 hrs, respectively. Scale bar: 20 μm.

Although many basic and clinical studies of ADSCs have demonstrated that the ADSCs transplantation could be considered as a promising therapeutic approach for various diseases^2^,^3^,^4^,^43^,^44^,^45^. Side-effects of ADSC therapy are greatly concerned throughout clinical applications, and it has been proved that pathological change was an indicator for treatment-induced toxicity^46^. Therefore, we evaluated the safety of the SPIONs cluster@PDAlabeled ADSCs transplantation. After 1 hr, 6 hrs and 24 hrs of the SPIONs cluster@PDA labeled ADSCs injection, the vital organs including heart, liver, spleen, lung and kidney were collected and histologically examined by using H&E staining, respectively. As shown in Fig. 7, there were not obvious pathological changes of the heart, liver, spleen, lung and kidney from all groups, which further confirmed the safety of SPIONs cluster@PDA-labeled ADSC transplantation.

## Conclusion

In summary, we demonstrated that SPIONs cluster@PDA nanoparticles could be used as a biocompatible MRI contrast agent for ADSCs tracking *in vivo*, without affecting their viability and proliferation, apoptosis, as well as the surface stem cell marker expression and the multi-differentiation potentials. The transplantation of labeled ADSCs into the mice through tail intravenous injection can home into the injured liver, and subsquentially repair the injured liver. Moreover, the enhanced homing capacity and therapeutic effects of the SPIONs cluster@PDA-labeled ADSCs could be achieved by use of external magnetic field in the excisional skin wound mice model. Therefore, our findings provide a noninvasive method for targeted delivery and tracking of transplanted cells into injured sites *in vivo*, which might provide new information and accelerate the clinical translations of the stem cell therapy.

## Methods

### Ethical statement

All experimental protocols were approved by Animal Ethics Committee of Mengchao Hepatobiliary Hospital of Fujian Medical University, and all experiments were carried out in accordance with the approved guidelines.

### Animals

Male ICR mice (4–5 weeks old; weighing:18–22 g) were obtained from the Center for Animal Experiment of Fujian Medical University (License No: SCXKmin2012-0002), and housed at constant temperature (22 ± 2 °C) and 60% relative humidity, with a light/dark (hours) cycles of 12/12.

### Isolate and Culture of ADSCs

For the isolation of ADSCs, the subcutaneous adipose tissues in groin were collected and cut into small pieces and digested with 0.1% type I collagenase (Sigma) in alpha modification of Eagle’s medium (α-MEM, Hyclone) at 37 °C for 60 min. Afterwards, the type I collagenase activity was neutralized with α-MEM containing 20% fetal bovine serum (FBS, Gibco), followed by filtering through a 100 μm cell strainer and washed in PBS by centrifugation at 400 × g for 10 min. Then, the cell pellet were resuspended with osmotic lysates (Biyuntian Biological Co.,Ltd, Shanghai, China) and incubated at room temperature for 10 min to lyse contaminating red blood cells. The remaining cells were seeded into T-75 flasks at a density of 1 × 10^6^/mL, and cultured with complete culture medium. Cells from the passage 3 or 4 were used in this study.

### Preparation of Polydopamine-coated SPIONs clusters (SPIONs clusters@PDA)

The superparamagnetic iron oxide nanoparticles (SPIONs) clusters were obtained as previous description^24^. Briefly, 1.2 mL of hydrophobic SPIONs nanoparticles (100 mg/mL in toluene) obtained by thermal decomposition method were sheared in 0.8 mL of 100 mM sodium dodecyl sulfate (SDS) aqueous solution by Branson Sonifier 250 at a duty cycle of 50% for 180 s. After that, the toluene was evaporated at 90 °C and the SPIONs clusters were collected by magnetic separation. To further coating a PDA layer on these clusters, 20 mg of the clusters was dispersed in 10 mL of dopamine solution (1 mg/mL in 10 mM of tris-HCl) containing 1.6 mM of SDS, and the mixture was continuously stirred at room temperature for 24 hrs. Finally, the SPION cluster@PDA was obtained by magnetic separation.

The morphology and size distribution of nanoparticles were examined by a transmission electron microscope (TEM, Tecnai G2 F20) operated at 200 kV. The dynamic size of nanoparticles was determined by dynamic light scattering (DLS) experiment which was performed at 25 °C on a NanoZS (Malvern Instruments, Malvern UK). Zeta potential measurements were performed at 25 °C on the NanoZS, using M3-PALS technology.

### Cell Viability Assay

Cells were cultured in a 96-well plate at 5 × 10^3^ cells/well, and incubated at 37 °C with 5% CO_2_ atmosphere for 24 hrs. The cell culture medium of each well was then replaced with 150 μL fresh complete medium containing SPIONs clusters@PDA at various Fe concentrations. Meanwhile, the cells incubated with cell culture medium only were prepared as untreated control. After incubation for 24 hrs or 48 hrs at 37 °C, cytotoxicity was performed by using a CCK-8 cell proliferation kit following the manufacture’s protocol (Dojindo Molecular Technologies, Tokyo, Japan). A microplate reader (Spectra Max M5) was used to measure the absorbance of the solution in each well at 450 nm. All experiments were performed in quadruplicate.

### Cell Apoptosis Assay

Flow cytometry (Becton Dickinson, USA) was used for cell apoptosis assay. Briefly, ADSCs were cultured in a 6-well plate at a density of 1 × 10^5^ cells/well for 24 hrs. Then, the culture medium was replaced with fresh complete medium containing 0.25 mM SPIONs clusters@PDA. After culture for 3 hrs, 6 hrs, 12 hrs and 24 hrs, cell apoptosis was performed using the Annexin V-FITC apoptosis assay kit (Dojindo Molecular Technologies, Tokyo, Japan), according to the manufacturer’s instructions.

TUNEL assay was also used to determine the cell apoptosis after incubated with SPIONs clusters@PDA. Similarly, the cells were cultured in a 24-well plate at a density of 3 × 10^4^ cells /well at 37 °C in a 5% CO_2_ atmosphere for 24 hrs. Next, cells were incubation with 0.25 mM SPIONs clusters@PDA dispersed in complete medium at 37 °C for 3 hrs, 6 hrs, 12 hrs and 24 hrs respectively. Afterwards, the cells were washed three times with PBS and fixed with 4% paraformaldehyde for 24 hrs. Then, TUNEL assay was performed using a Situ Cell Death Detection Kit (Roche Applied Sciencem, Mannheim, Germany) following manufacture’s protocol, then the cells were visualized by an inverted fluorescence microscope (Zeiss, Germany).

### Cell phenotypes analysis

To investigate the effect of SPIONs clusters@PDA on cell phenotypes of ADSCs, cells were seeded at a density of 1 × 10^5^ cells/well and allowed to attach for 24 hrs. Next, cells were treated with or without 0.25 mM SPIONs clusters@PDA for another 24 hrs. Then, the unlabeled and labeled cells were trypsinized and suspended with PBS containing 5% bovine serum albumin (BSA, Sigma, USA); afterwards, the cells were incubated with different primary antibodies as indicated for 60 min at room temperature, including CD29-PE and CD44-PE (eBioscience, CA), CD31, CD34, CD45, CD73 and CD90 (Santa Cruz Biotechnology, USA), as well as CD105 and HLA-DR (Abcam, UK), respectively. Subsequently, the cells were washed with PBS for twice, and then incubated with the corresponding fluorescent conjugated secondary antibodies (donkey anti-mouse IgG- Alexa fluor 488 or donkey anti-rabbit IgG-alexa fluor 647, Invitrogen, USA) for 30 min at room temperature. Finally, the cells were characterized by flow cytometry.

### Multi-differentiation potential analysis

To assess the effects of SPIONs clusters@PDA on the ability of ADSCs to differentiate into multi-lineages, ADSCs were seeded into 12-well plates at a density of 3 × 10^4^ cells/well in 1 mL complete medium. After reaching 80% confluence, the cultured cells were treated with or without 0.25 mM SPIONs clusters@PDA for 24 hrs. Afterwards, the cultured cells were inducted by the osteogenic medium, adipogenic medium and chondrogenic medium respectively, and the induction medium was changed every 3 days. The composition of induction medium was described previously^47^, and all chemicals were purchased from Sigma-Aldrich. For osteogenesis, cells were cultured in osteogenic induction medium consisting of basic growth medium (α-MEM plus 10% FBS) added with 0.1 mM dexamethasone, 50 mM ascorbate-2-phosphate and 10 mM β-glycerol phosphate; after 4 weeks of culture, cells were fixed with 4% paraformaldehyde for 60 min at room temperature, washed with distilled water for twice and stained with 0.1% Alizarin Red S solution (Solarbio, Beijing, China) for 30 min. For adipogenesis, cells were cultured in adipogenic induction medium consisting of basic growth medium (α-MEM plus 10% FBS) added with 0.5 mM isobutyl-methylxanthine (IBMX), 1 mM dexamethasone, 10 mM insulin and 200 mM indomethacin; after 2 weeks of culture, cells were stained by an Oil Red O staining kit (Nanjing Jiancheng Bioengineering Institute, Nanjing, China) following manufacture’s protocol. For chondrogenesis, cells were cultured in chondrogenic induction medium consisting of basic growth medium (α-MEM plus 1% FBS) added with 6.25 mg/mL insulin, 10 ng/mL TGF-β1 and 50 nM ascorbate-2-phosphate; after 4 weeks of culture, cells were stained by a toluidine blue staining kit (Nanjing Jiancheng Bioengineering Institute, Nanjing, China) following the manufacture’s protocol.

### Quantitative Real-time PCR analysis

To evaluate the effects of SPIONs clusters@PDA on the expression of the stem cell makers of ADSCs, Nanog, octamer-binding transcription factor 4 (OCT-4), SRY (sex determining region Y)-box 2 (SOX-2) and C-X-C chemokine receptor type 4 (CXCR-4) were examined by Quantitative Real-time PCR analysis. After incubation the ADSCs with 0.25 mM SPIONs clusters@PDA for 24 hrs, the incubated cells were collected and the total RNA was extracted using TRIzol reagent (TransGen Biotech, Beijing, China). Next, the mRNA was reversely transcribed to cDNA by a Transcriptor First Strand cDNA Synthesis Kit (Roche Applied Sciencem, Mannheim, Germany), and quantitative real-time PCR analysis was performed using the ABI step one plus RealTime PCR System (Carlsbad, CA, USA) with SYBR Green PCR Master Mix (Toyobo, Osaka, Japan). Cycling conditions were as follows: 40 cycles of 95 °C for 15 s, 60 °C for 30s, and 70 °C for 30 s. The PCR primers were as follows: Nanog forward primer, 5′-AGATCTCGCCTGTTACAGTTCTTTG-3′ and reverseprimer, 5′-AGTCCAGCTGGCACTGGTTT-3′; OCT-4 forward primer, 5′-CCACACTCTACTCGGTCCCTTT-3′ and reverse primer, 5′-GGTGCCTCAGTTTGAATGCA-3′; SOX-2 forward primer, 5′-GAGTAAGAAAAATCTGAATGCTCAA-3′ and reverse primer, 5′-AGCGCCTAACGTACCACTAGAA-3′; CCR-4 forward primer,5′-GGTCTGGAGACTATGACTCCA-3′ and reverse primer, 5′-GTGCTGGAACTGGAACACCA-3′; β-actin forward primer, 5′-GGAGATTACTGCCCTGGCTCCTA-3′ and reverse primer, 5′-GACTCATCGTACTCCTGCTTGCTG-3′. The expression of target gene was normalized to that of β-actin gene. Relative gene expression was calculated with the 2^−△Ct^ formula.

### Evaluation of Cellular Uptake by Prussian blue staining

ADSCs were cultured in a 6-well plate at a density of 1 × 10^5^ cells/well, and incubated with 0.25 mM SPIONs clusters@PDA for 3 hrs, 6 hrs, 12 hrs and 24 hrs, respectively. At the indicated time point, the cells were washed with PBS, and followed by fixation with 4% paraformaldehyde. Subsequently, the cells were washed with deionized water and incubated with fresh prepared Perls reagent (4% potassium ferrocyanide /12% HCl, 1: 1, v/v) for 30 min at 37 °C. Afterwards, the cells were washed with PBS and imaged by an inverted microscope (Zeiss, Germany).

### Quantification of Iron Content

Since Fe^3+^ can form a highly colored complex after reacted with thiocyanate ion, a colorimetric method was used to study the iron concentration of SPIONs cluster or intracellular iron content^24^. 50 μL of sample solution which was dissolved in 12% HCl was added into the well of a 96-well plate, and then 50 μL of 1% ammonium persulfate was added to oxidize the Fe^2+^ to Fe^3+^. Finally, 100 μL of 0.1 M potassium thiocyanate was added to the well and incubated for 5 min to form the red iron-thiocyanate. The absorption was read by a microplate reader at a wavelength of 490 nm.

For the intracellular iron content study, ADSCs were seeded in a 6-well plate at a density of 1 × 10^5^ cells/well, and incubated with 0.25 mM SPIONs clusters@PDA for 3 hrs, 6 hrs, 12 hrs and 24 hrs. After the incubation, the cells were washed with PBS and then trypsinized and dispersed in 1 mL of distilled water. 500 μL of cell suspension was taken out to determine the concentration of iron by a colorimetric method mentioned above. Cells which were not incubated with SPIONs clusters@PDA were also collected and measured as a control. Subsequently, the readout value was normalized against the corresponding protein concentration of the residual 500 μL of cell suspension, which was determined by a bicinchoninic acid (BCA) assay kit (TransGen Biotech, Beijing, China).

### Cells Imaging with Magnetic Resonance

ADSCs were seeded at a density of 5 × 10^4^ cells/cm^2^ into the T-25 flasks overnight to allow cell attachment, followed by treatment with 0.25 mM SPIONs clusters@PDA nanoparticles for 3 hrs, 6 hrs, 12 hrs and 24 hrs. After the incubation, cells were washed with PBS for three times, then harvested and embedded into 1 mL 1% (w/v) agarose. Finally, the cells were imaged using a 7.0 T small-animal MRI scanner (Bruker Avance II 500WB spectrometer). Cells which were not incubated with SPIONs cluster@PDA were also collected and imaged as a control.

### Animal Models and the Transplantation of SPIONs cluster@PDA labeled ADSCs

The procedures for developing a carbon tetrachloride (CCl_4_) induced liver injury model and excisional skin wound model were performed according to the previously described protocols with minor modification^48^,^49^. Briefly, For the CCl_4_ induced liver injury model, male ICR mice (4–5 weeks) were injected intraperitoneally with a single dose of 2% CCl_4_ solution in olive oil (10 mL/kg body weight). For excisional skin wound model, male ICR mice (4–5 weeks) were anesthetized with 2% pentobarbital sodium (40 mg/kg body weight, ip) and followed by hair removal from the dorsal surface; subsequently, a 6-mm full thickness excisional skin wound was created on right side of the midline. After 24 hrs, all animal models were treated with tail vein injection of 1 × 10^6^ SPIONs cluster@PDA labeled ADSCs (in 0.1 mL PBS), which were prepared by incubating in α-MEM containing 10% FBS and 0.25 mM SPIONs cluster@PDA for 24 hrs. After cell injection, the excisional skin wound animals were subsequently fastened with a magnet to investigate the external magnetic field guided target-cell homing to the wound and the repair effects of labeled ADSCs. All mice were imaged at the indicated time points.

### *In vivo* MRI Imaging

The *in vivo* MR imaging was conducted in a 7.0 T horizontal bore small animal MRI scanner (Bruker, Germany). Briefly, all mice were anesthetized with 1–2% isoflurane mixed with pure oxygen via a nose cone, and were placed in a stretched supine position coupled with a micro-mouse RF probe (Bruker, Germany). *In vivo* T_2_-weighted MR images were acquired using rapid acquisition with relaxation enhancement (RARE) sequence. Imaging parameters were Repetition Time (RT), 3000 ms, Echo Time (ET), 33 ms, Rare Factor, 8, Field of View (FOV) = 50 × 50 mm^2^, image size, 256 × 256, slice thickness, 0.5 mM and number of average, 6.

### Recombinant lentivirus production

HEK-293T cells were cultured in the 10 cm plate (1 × 10^6^ cells/plate) in Dulbecco’s Modified Eagle’s Medium (DMEM) (Gibico) with 10% Fetal bovine serum (FBS) (Gibico), and incubated at 37 °C with 5% CO_2_ atmosphere for 24 hrs. Then, the medium was replaced with fresh culture medium 2 hrs before transfection. Lipofectamine 3000 (Life technologies) was used to transfect the cells following the manufacture’s instruction, and 10 μg of pCDH-CMV-MCS-EF1-puro-EGFP plasmids was used for one 10 cm dish. Transfection medium was replaced by fresh culture medium after 16 hrs of transfection. Medium containing the viruses was harvested after 48 and 72 hrs of transfection and centrifugated at 3,000g for 30 min, then filtered through a 0.45 μm filter before transduction.

### ADSCs transduction by lentivirus

Second passage ADSCs were cultured in six-well plates at the density of 1 × 10^5^ cells/well. After 24 hrs, 10 MOI (multiplicity of infection) fresh prepared recombinant lentivirus per well supplemented with 5 μg/mL polybrene (Sigma Aldrich) were added into the ADSCs cells. After 24 hrs of lentiviral transduction, the medium of cells was replaced by fresh culture medium, and the ADSCs cells were harvested for further usage.

### Fluorescence imaging

The male ICR mice, which were injured by a single dose of 2% CCl_4_ solution in olive oil (10 mL/kg body weight) for 24 hrs, were applied as liver injury model. The mice, which were intravenously transplanted with the EGFP-transduced ADSCs (1 × 10^6^ cells per mouse), were used for ex vivo fluorescence imaging analysis. After certain time of transplantation, major organs (heart, lung, kidney, spleen and liver) were harvested and immediately subjected to fluorescence imaging using an IVIS Series Pre-clinical *In Vivo* Imaging Systems (PerkinElmer) (excitation filter: 490 nm ± 10 nm; emission filter: 525 nm ± 10 nm).

### Histo-pathological assessment

To investigate the toxicity of the transplantation of SPIONs cluster@PDA labeled ADSCs *in vivo*, the normal male ICR mice (4–5 weeks) were transplanted with tail vein injection of 1 × 10^6^ SPIONs cluster@PDA labeled ADSCs (in 0.1 mL PBS) for 1 hr, 6 hrs, and 24 hrs respectively. Afterwards, the main organs including heart, liver, spleen, lung and kidney were collected for histo-pathological assessment. Briefly, the obtained tissues were fixed in 4% paraformaldehyde for 24 hrs, then gradually dehydrated with ethanol and embedded in paraffin. Subsequently, tissue sections of 5 μm thickness were stained with hematoxylin and eosin (H&E) for histological analysis.

To further observe the homing and therapeutic effects of the SPIONs cluster@PDA labeled ADSCs on liver injury *in vivo*, the liver tissues of CCl_4_ induced liver injury mice which transplanted with 1 × 10^6^ SPIONs cluster@PDA labeled ADSCs (in 0.1 mL PBS) via tail vein injection, were also collected for histo-pathological assessment as the aforementioned steps. For the observation of the hepatic homing, the liver sections of 5 μm thickness were stained by a by a Prussian blue staining kit (Shanghai Yuanye Biological Technology co., LTD, Shanghai, China) following the manufacture’s protocol. The histopathological examination was performed by using a Zeiss microscope (Lab.A1, Germany), and were double blind evaluated by 2 pathologists.

### Statistical analysis

All quantitative data were expressed as the mean ± standard deviation (SD). Graph Pad Prism version 6.0 was used for statistics analysis. Statistical analysis among different groups was performed using student T-Test. The p < 0.05 was considered as statistically significant.

## Additional Information

**How to cite this article**: Liao, N. *et al.* Poly (dopamine) coated superparamagnetic iron oxide nanocluster for noninvasive labeling, tracking, and targeted delivery of adipose tissue-derived stem cells. *Sci. Rep.* **6**, 18746; doi: 10.1038/srep18746 (2016).

## Supplementary Material

Supplementary Information
