# Supplementary material for: Poly (dopamine) coated superparamagnetic iron oxide nanocluster for noninvasive labeling, tracking, and targeted delivery of adipose tissue-derived stem cells
Source: Sci Rep. 2016 Jan 5;6:18746. doi: 10.1038/srep18746 (PMC4700528; doi:10.1038/srep18746)
Supplement: Supplementary Information [file srep18746-s1.doc]

Supplementary information for “Poly (dopamine) coated superparamagnetic iron oxide nanocluster for noninvasive labeling, tracking, and targeted delivery of adipose tissue-derived stem cells”

Naishun Liao^1, 2,a^, Ming Wu^1, 2,a^, Fan Pan^3^, Jiumao Lin^4^, Zuan fang Li^4^, Da Zhang ^1, 2^, Yingchao Wang^1, 2^, Youshi Zheng^1, 2^, Jun Peng^4^, Xiaolong Liu^1, 2,*^, Jingfeng Liu^1, 2, 5,*^

^1^ The United Innovation of Mengchao Hepatobiliary Technology Key Laboratory of Fujian Province, Mengchao Hepatobiliary Hospital of Fujian Medical University, Fuzhou 350025, P.R. China

^2^ The Liver Center of Fujian Province, Fujian Medical University, Fuzhou 350025, P.R. China

^3^ Department of Hepatobiliary Surgery, Fuzong Clinical College, Fujian Medical University, Fuzhou 350001, P.R. China

^4^ Academy of Integrative Medicine, Fujian University of Traditional Chinese Medicine, Fuzhou 350122, P.R. China

^5^ Liver Disease Center, The First Affiliated Hospital of Fujian Medical University, Fuzhou 350007, P.R. China

*** Corresponding Author (correspondence should be address to Xiaolong Liu and Jingfeng Liu); Mengchao Hepatobiliary Hospital of Fujian Medical University, Fuzhou, Fujian 350025, P. R. China. Tel: +86 591 83705927. Fax: +86 591 83705927. E-mail addresses: xiaoloong.liu@gmail.com, drjingfeng@126.com

^a^ These authors contributed equally to this work.


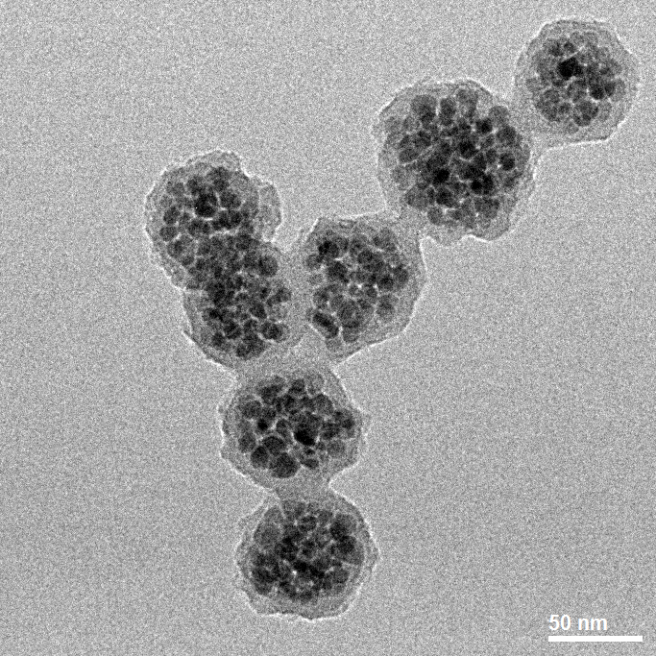


Figure S1. **TEM image of SPIONs cluster@PDA.**

**
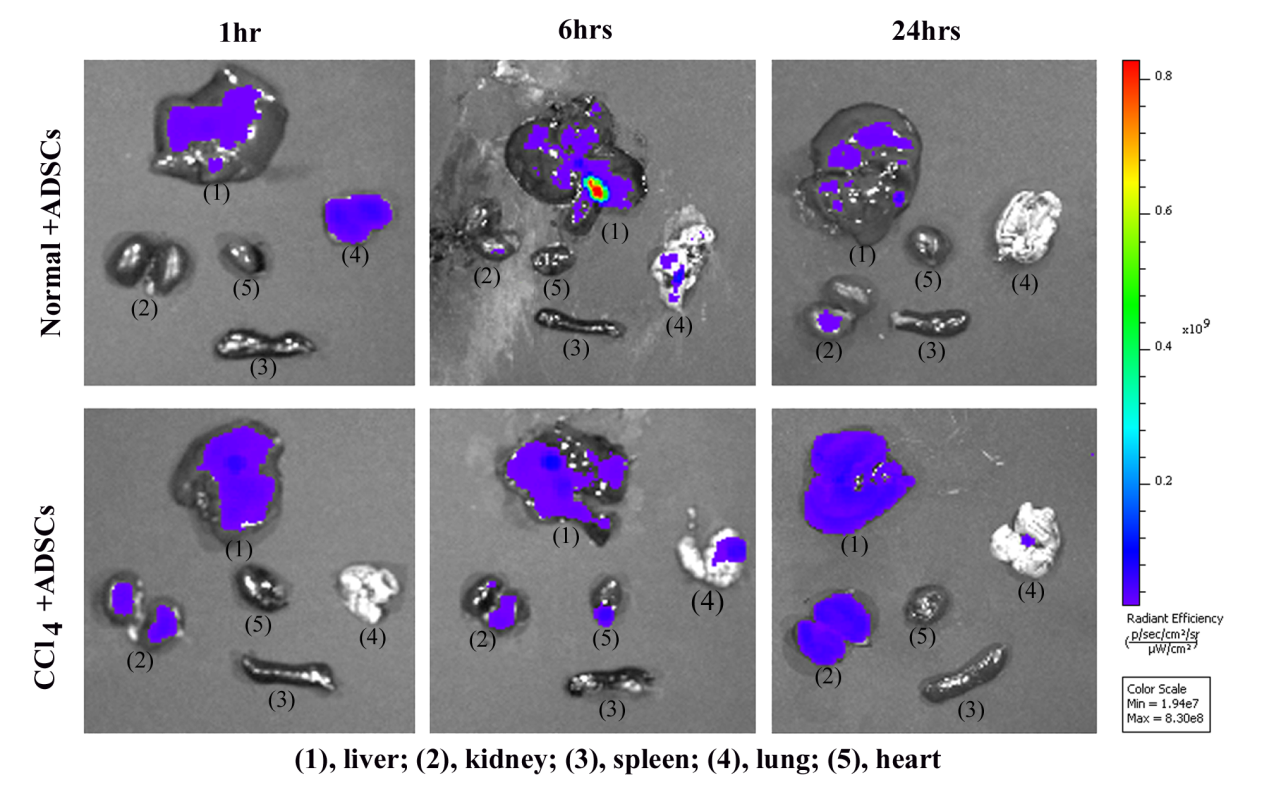
**

Figure S2. **Fluorescence images of major organs harversted from the mouse after 1hr, 6hrs or 24hrs of transplantation with the ADSCs, which are transduced by a pCDH-CMV-MCS-EF1-puro-EGFP lentiviral vector.**
